# Transcriptome Functional Analysis of Mammary Gland of Cows in Heat Stress and Thermoneutral Condition

**DOI:** 10.3390/ani10061015

**Published:** 2020-06-10

**Authors:** Shuangming Yue, Zhisheng Wang, Lizhi Wang, Quanhui Peng, Bai Xue

**Affiliations:** Animal Nutrition Institute, Sichuan Agricultural University, Chengdu 611130, China; yueshuangming@stu.sicau.edu.cn (S.Y.); wangzs@sicau.edu.cn (Z.W.); 12825@sicau.edu.cn (L.W.); 14101@sicau.edu.cn (Q.P.)

**Keywords:** heat stress, dairy cow, whole transcript sequencing, immune response, stress response

## Abstract

**Simple Summary:**

The current study employed RNA-seq technology to analyze the impact of heat stress on the whole transcript sequencing profile in the mammary glands of lactating Holstein dairy cows. In the findings of the current study, heat stress downregulated the expression of casein genes, which resulted in a decrease in milk production. Moreover, heat stress upregulated the gene expression of *HSPA1A* and *HSP90B1*, while it downregulated the expression of immune response-related genes that resulted in a reduction in milk yield. Furthermore, there was an increased synthesis of heat shock proteins and unfolded proteins that could reduce the availability of circulating amino acids for milk protein synthesis. The findings of the current experiment may help to explore the impact of heat stress on immune function, milk production, and milk protein synthesis in cows.

**Abstract:**

Heat stress (HS) exerts significant effects on the production of dairy animals through impairing health and biological functions. However, the molecular mechanisms related to the effect of HS on dairy cow milk production are still largely unknown. The present study employed an RNA-sequencing approach to explore the molecular mechanisms associated with a decline in milk production by the functional analysis of differentially expressed genes (DEGs) in mammary glands of cows exposed to HS and non-heat-stressed cows. The results of the current study reveal that HS increases the rectal temperature and respiratory rate. Cows under HS result in decreased bodyweight, dry matter intake (DMI), and milk yield. In the current study, a total of 213 genes in experimental cow mammary glands was identified as being differentially expressed by DEGs analysis. Among identified genes, 89 were upregulated, and 124 were downregulated. Gene Ontology functional analysis found that biological processes, such as immune response, chaperone-dependent refolding of protein, and heat shock protein binding activity, were notably affected by HS. The Kyoto Encyclopedia of Genes and Genomes enrichment analysis found that almost all of the top-affected pathways were related to immune response. Under HS, the expression of heat shock protein 90 kDa beta I (*HSP90B1*) and heat shock 70 kDa protein 1A was upregulated, while the expression of bovine lymphocyte antigen (*BoLA*) and histocompatibility complex, class II, DRB3 (*BoLA-DRB3*) was downregulated. We further explored the effects of HS on lactation-related genes and pathways and found that HS significantly downregulated the casein genes. Furthermore, HS increased the expression of phosphorylation of mammalian target of rapamycin, cytosolic arginine sensor for mTORC1 subunit 2 (*CASTOR2*), and cytosolic arginine sensor for mTORC1 subunit 1 (*CASTOR1*), but decreased the phosphorylation of Janus kinase-2, a signal transducer and activator of transcription factor-5. Based on the findings of DMI, milk yield, casein gene expression, and the genes and pathways identified by functional annotation analysis, it is concluded that HS adversely affects the immune function of dairy cows. These results will be beneficial to understand the underlying mechanism of reduced milk yield in HS cows.

## 1. Introduction

In livestock production, heat stress (HS) negatively affects livestock health and production [1]. However, the effects of HS on livestock production are different in different livestock breeds [2,3]. For example, HS in beef breeds is generally believed less critical as compare to dairy breeds because beef breeds have lower metabolic rates and lower body heat production [2,3]. Therefore, most of the studies explore the impact of HS on dairy animals’ health and production.

In dairy animals, the Holstein breed is one of the standard commercial dairy cattle breeds and is widely known due to its high milk yield. In hot and humid seasons, the ability of Holstein dairy cattle to dissipate body heat via skin evaporation is restrained due to its relatively low surface area to body weight ratio, dense body surface hair, and underdeveloped sweat glands [4]. Therefore, Holstein dairy cattle are at higher risk of facing even more severe heat stress [5]. Previous studies have reported that HS negatively influences the production of milk and protein contents of milk in Holstein dairy cows [6,7]. It is traditionally believed that HS results in lower dry matter intake (DMI), which reduces the production of milk yield and protein contents of milk in dairy cows [8,9]. However, the utilization of pair-fed thermal neutral (TN) controls in recent studies have demonstrated that decreased DMI only partially (about 35–50%) explains the decrease in productivity [6,7]. It has also been reported that lower DMI in HS cows is not only one of the main reasons for the decline in milk production [6,7]. Furthermore, it has also been reported that HS influences the cellular response that is responsible for the decline in milk production and milk quality [10]. For example, Cowley et al. [7] found that HS results in a reduction in milk protein as a result of downregulation of bovine mammary epithelial cell (BMEC) activity [7]. Moreover, at the cellular level, heat stress adversely affects the function and gene expression of casein in BMECs [11,12], especially the Alpha casein S1 (*CSN1S1*) [13]. The in-vitro studies explored that high ambient temperature downregulates the gene expression involved in cell structure, biosynthesis, and transport, whereas it upregulates the gene expression involved in protein repair and degradation in BMECs [7,14].

However, the effect of HS on the whole transcript sequencing in mammary tissue of dairy cows in-vivo is still unknown. Therefore, the current experiment was designed to investigate the global expression profile of dairy cows’ mammary gland tissue during normal and HS state and to identify the molecular pathways regulated in heat-stressed dairy cows by using RNA sequencing (RNA-Seq). The objective of the current study is to explore the effect of HS on the molecular mechanism that reduces the performance of dairy cows.

## 2. Materials and methods

### 2.1. Animals, Management and Experimental Treatments

All experimental protocols in the current study were conducted following the guidelines of the Animal Care and Use Committee, constituted by Sichuan Agricultural University (Chengdu, China). The current experiment was conducted at Qingbaijiang Dairy Farm of New Hope Dairy Co., Ltd. (Chengdu, China). A total of twenty Holstein cows, with healthy and symmetrical udders, were selected for this study. All the experimental cows were reared in a closed-type cowshed to avoid seasonal variation and photoperiods on metabolism. Individual pens were assigned to each cow in such a way that each cow had free access to fresh drinking water. The managemental regimes were the same for all the experimental cows. A group of ten cows was considered for HS experimental treatment, while the second group of 10 cows was considered for TN experimental treatment. In both experimental groups, each cow was considered as a replicate. The basic information of experimental cows is shown in Table 1.

The samples of HS treatment were collected in the summer season (from mid-July to late August in 2018) when the environmental temperature–humidity index (THI) was enhanced from 72.5 to 86.9 over one month and stabled at 80.5 for one week. The samples of TN experimental treatments were collected in the spring season (from mid-March to late April in 2018) when environmental THI steadily increased from 52.1 to 65.2 over a one-month period. Experimental cows were on the same diet throughout the trial period. The duration of the adaptation period was 15 days, while the duration of the experimental data collection was 30 days.

Animals were fed the diet in the form of a total mixed ration (TMR). The delivery of TMR was at 07:00, 14:00, and 20:00 h of the day throughout the experimental period. The diets of experimental animals were formulated according to the recommendation of National Research Council (NRC 2001) for dairy cows [15]. The experimental feed ingredients profile and chemical composition are shown in Table 2.

In the current experiment, relative humidity and temperatures were recorded on a daily basis. The time of recording of temperatures and relative humidity was 07:00, 14:00, and 20:00 h of the day. A hygrometer and a thermometer on one instrument panel (Jiangsu JingChuang Electric Co. Ltd., Nanjing, China) were used for the determination of relative humidity and temperatures. The temperature-humidity index was calculated according to NRC.1971.

THI = 1.8 × T + 32 − 0.55 × (1 − RH) × (T × 1.8 + 32 − 58)(1)

where T.was the ambient temperature determined by the dry bulb in °C. and RH was the relative humidity in %.

Bodyweight (BW), rectum temperature (RT), respiratory rate (RR), and milk quality indexes were recorded or determined for all experimental cows. Respiratory rate and RT were measured three times a day at 07:00, 14:00, and 20:00 h. Respiratory rate was calculated by counting the total number of flank movements/min for 120 s. Rectal temperature was obtained with a GLA 525/550 digital thermometer.

### 2.2. Sample Collection

On the last day of the experimental period, blood samples (duplicate samples) were taken from the median coccygeal vein of each cow before first feeding (morning). Blood was collected into evacuated tubes with and without anticoagulant. The samples of blood were kept at a cool place until they were centrifuged for 15 min at 3000× *g* (4 °C) to separate serum or plasma. Obtained serum and plasma were stored at −80 °C for further analysis. The concentrations of heat shock protein 70 (HSP70) and lipopolysaccharide (LPS) in plasma were determined using an ELISA kit (Beyotime Biochemical Reagent Co., Shanghai, China). Furthermore, the serum obtained was used to analyze nonesterified fatty acid (NEFA) and glucose concentration by automatic biochemical analyzer 7600 (Hitachi, Tokyo, Japan).

After a 3-week rearing of experimental cows in either HS or TN group, three cows with the same average milk yield were chosen for taking mammary gland samples. The biopsy procedure was carried out according to established methods, as described in the previous study [16]. Biopsies were operated after approximately 6 h of milk accumulation. To carry out the biopsy procedures, experimental cows were properly restrained, and an intravenous injection of xylazine hydrochloride (35–45 μg/mg of BW, romazine 2%, Healton Animal Health, Neijiang, China) was applied. A 10-cm^2^ area of udder skin on the right rear quarter was clipped, cleaned, and sterilized. The area for biopsy was anesthetized by injection (subcutaneous) of 3 mL of lignocaine hydrochloride (20 mg/mL. of lopaine, Healton Animal Health, Neijiang, China). A 1–2 cm incision was made through the skin and gland capsule. The incision was made in such a way to avoiding any large subcutaneous blood vessels.

The biopsy instrument (Wuhan Anscitech Farming Technology, Wuhan, China) was used to cut a core (70 × 4 mm. in diameter) of mammary tissue. To control bleeding, we inserted a 3 × 5 cm surgical plug (Healton Animal Health, Neijiang, China) into the wound. After that, Michel suture clips were used to close the skin incision. Antibiotic powder was also applied onto the wound (terramycin powder oxytetracycline hydrochloride (2% wt/wt), North China Pharmaceutical Group Corporation Veterinary, Shijiazhuang, China). A single intramuscular dose of penicillin and streptomycin (4 mL/1000 kg of BW, North China Pharmaceutical Group Corporation Veterinary, Shijiazhuang, China) was also given instantly after the biopsy. After the biopsy, the cows were machine milked. To remove intramammary blood clots, hand-stripping was used. Furthermore, cows were hand-stripped as required at each milking for the next 4–7 days until all blood clots were removed entirely. Michel suture clips were removed 7–10 days after the biopsy. In the subsequent duration of the experiment, after the first milking, both rear glands received a prophylactic dose of intramammary antibiotic (200 mg of sodium cloxacillin, North China Pharmaceutical Group Corporation Veterinary, Shijiazhuang, China). The same intramammary antibiotic dosage was repeated after every two days. Representative tissues of the mammary gland were sampled, weighed, washed by cold phosphate-buffered saline, and then kept in liquid nitrogen until further analysis.

### 2.3. RNA Isolation and Library Preparation

Total RNA was extracted from the mammary gland tissue of cows of the HS group and the TN group by utilizing trizol reagent (Invitrogen, South San Francisco, CA, USA). The manufacturer’s protocol was strictly followed to obtain total RNA. A NanoDrop 2000 spectrophotometer (Thermo Scientific Scientific, Inc., Waltham, MA, USA) was used to evaluate RNA purity and quantification. Furthermore, to evaluate RNA integrity, an Agilent 2100 Bioanalyzer (Agilent Technologies, Santa Clara, CA, USA) was used. Samples with an RNA Integrity Number (RIN) >7 were further subjected for analysis. The libraries were constructed by employing the TruSeq Stranded mRNA LTSample Prep kit (Illumina, San Diego, CA, USA) by following the manufacturer’s protocol. Then, these libraries were sequenced on an Illumina HiSeq X Ten platform (OE Biotech Co., Ltd, Shanghai, China), and 150-bp paired-end reads were generated.

### 2.4. Quality Control and Mapping

Raw reads were generated from the images by using Base Calling, and the quality of the raw reads was checked by using Trimmomatic (San Diego, CA, USA). The low-quality reads and those containing poly-N were removed [17]. Then, the clean reads were mapped to the cow genome (*Bos taurus*) by using Hisat2 [18]. 

### 2.5. RNA-Seq Data Analysis

The FPKM (fragments per kb per million reads) value of each gene was measured by using cufflinks [19]. Then, HTSeq-Count was used to obtain the read counts of each gene. Differentially expressed genes (DEGs) were analyzed using the DESeq R package [20]. The corrected *p* < 0.05 and fold change (FC) > 1.5 or fold change (FC) < 0.67 was set as the thresholds for significantly differential expression. A hierarchical cluster analysis of DEGs was performed to examine gene expression patterns. The DEGs were annotated by Gene ontology (GO) functional enrichment and Kyoto Encyclopedia of Genes and Genomes (KEGG) pathway enrichment using the R programming language (3.5 versition, http://www.r-project.org/), based on the hypergeometric distribution.

### 2.6. Quantitative Real-Time PCR Analysis (qRT-PCR).

To verify the expression of DEGs identified by the RNA-seq approach, four DEGs, including *HSP70*, *HSP90B1*, bovine lymphocyte antigen (*BoLA*), and major histocompatibility complex, class II, DRB3 (*BoLA-DRB3*), were randomly picked for real-time PCR analysis. Moreover, 10 genes involved in lactation were selected for RT-PCR analysis, including *CSN1S1*, *CSN2, CSN3*, *PRLR*, STAT5A, *STAT5B*, *CASTOR1*, *CASTOR2*, *mTOR*, and *JAK2*. The PrimeScript™ RT reagent Kit (TaKaRa, Kyoto, Japan) was used to synthesis cDNAs in qRT-PCR from the same RNA extractions used for the RNA-seq. Furthermore, cDNAs were diluted 1:5. Glyceraldehyde-3 phosphate dehydrogenase (*GAPDH*) was selected as the control gene. qRT-PCRs were carried out in an ABI 7500 real-time thermocycler (Applied Biosystems, Foster City, CA, USA). The primers used are listed in Supplementary Materials Table S1. The comparative cycle threshold (2^−ΔΔCt^) method was used to determine the relative gene expression. Data are expressed as mean.±.standard error of the mean and were analyzed using SAS 9.2 software (SAS Institute Inc, Cary, NC, USA).

### 2.7. Western Blot Analysis

Mammary gland tissues were solubilized in RIPA Lysis and Extraction Buffer (Invitrogen; Thermo Fisher Scientific, Inc., Waltham, MA, USA) to extract total protein. After boiling for 5–10 min, the protein samples extracted from different cell suspensions were separated by SDS-PAGE and then transferred onto a nitrocellulose membrane. The membrane was blocked with 5% nonfat dry milk prepared in Tris-buffer and incubated with the primary antibodies (Santa Cruz Biotechnology, Inc., Santa Cruz, CA, USA) for 1 h at room temperature. The membrane was then incubated with a horseradish peroxidase-conjugated anti-rabbit IgG secondary antibody (Santa Cruz Biotechnology, Inc., Santa Cruz, CA, USA), for 4 h at room temperature. The blot was developed using the ECL™ Western Blotting Detection Reagent (GE Healthcare, Piscataway, NJ, USA), and the proteins were visualized by enhanced chemiluminescence (Amersham Biosciences, Piscataway, NJ, USA).

### 2.8. Statistical Analysis 

Data for DMI, milk yield, milk composition, somatic cell count (SCC), HSP70, LPS, glucose, NEFA, RR, and RT were analyzed by the *t*-test (SAS 2003, ver. 9.2, Inst. Inc., Cary, NC, USA). Each individual cow was considered as an experimental unit. Statistical significance was set at *p* < 0.05.

## 3. Results

### 3.1. Temperature–Humidity Index and Physiological Index

**Figure 1:**
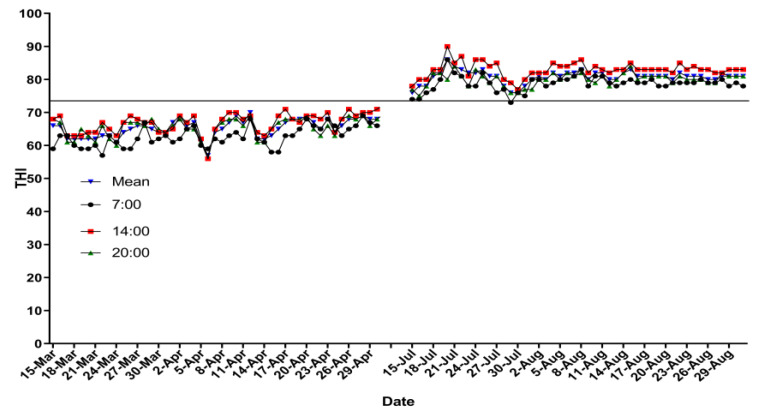
Temperature–humidity index at 07:00, 14:00, and 20:00 h, and mean.

The mean daily THI ranged from 55.6 to 63.2 during the spring period (averaged 59.8 in the whole period). The mean daily THI was from 79.0 to 83.2 during the summer period (averaged 79.5 in the whole period). The details of THI in both experimental treatments are presented in Figure 1. Respiratory rate and RT were higher (*p* < 0.01) in the HS experimental group than those in the TN experimental group (Table 3).

### 3.2. Effect of Exposure of Heat Stress on Milking Performance and Blood Biochemical Indexes

The results of milk production, milk composition, and blood biochemical indexes are presented in Table 4. Milk production decreased in animals of the HS group (*p* < 0.01) as compared to the TN group animals. Results also revealed a dramatic loss of BW and DMI in the animals of the HS group (*p* < 0.01). Both protein and fat in milk were decreased in the HS group animals (*p* < 0.05). In addition, the concentrations of plasma LPS, HSP70, and NEFA were enhanced, whereas the level of glucose was reduced in the HS group (*p* < 0.05).

### 3.3. HS Group Changed the Transcriptome in Mammary Gland Tissues 

**Figure 2:**
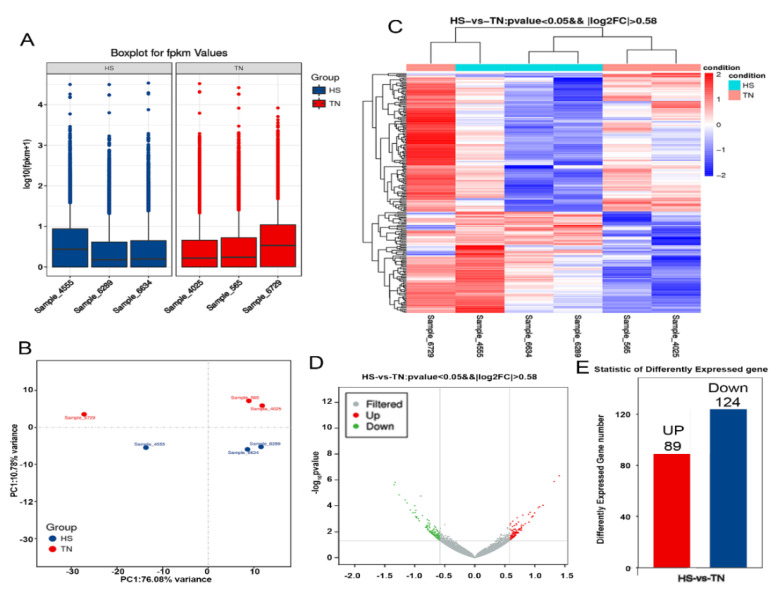
Effect of HS on transcriptomes in mammary gland tissues. (**A**) The distribution of FPKM (fragments per kb per million reads) values in HS and TN groups. (**B**) Principal component analysis was utilized to determine the reliability of the data, simplify the complexity of RNA-seq, and reduce the effective dimension of gene expression space. Heatmap (**C**) and volcano map (**D**) show the differentially expressed genes between HS and TN groups. (**E**) A total of 213 differentially expressed genes (DEGs) were identified, among which 89 were upregulated, and 124 downregulated between the groups studied.

An average of 49.30 million raw reads was generated from TN and HS libraries. After filtering out low-quality sequences, we had 47.4 to 48.76 million total tag numbers per library. High-quality (Q > 30) reads accounted for 95.27–96.04% of the reads and the GC percentage of each library ranged from 46.90% to 49.24% (Supplementary Materials Table S2). In order to evaluate the transcript profiling data, principal component analysis (PCA) was applied to capture the overall variance among the samples in two dimensions (Figure 2B). The principal component analysis illustrated that a significant difference existed between the TN and HS groups. Two hundred and thirteen DEGs were identified, among which 89 were upregulated and 124 downregulated (Figure 2E). The DEG profiles in six samples are shown in Figure 2C,D.

### 3.4. Gene Ontology Enrichment Analysis

**Figure 3:**
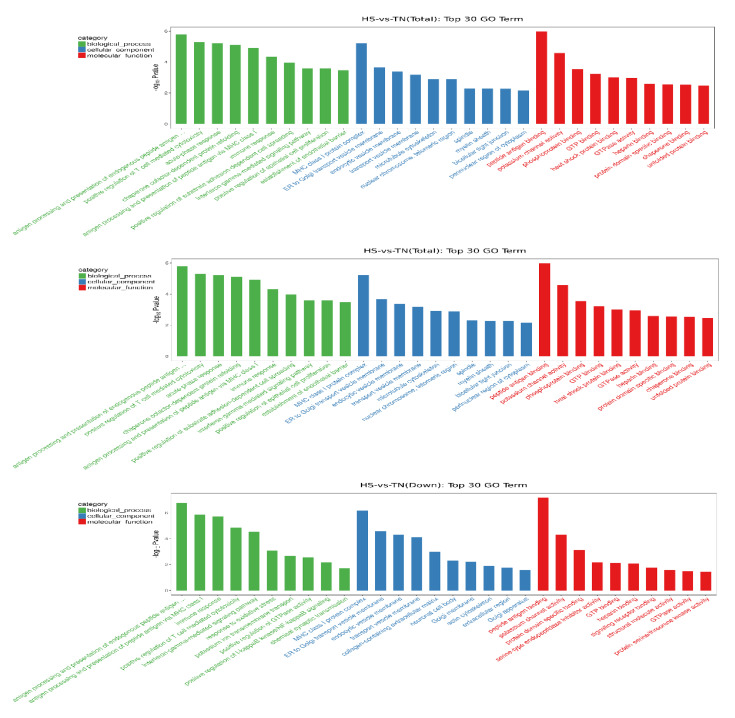
Gene ontology enrichment analysis. The genes from hierarchical clustering were further analyzed with the Database for Annotation Visualization and Integrated Discovery. The categories of the most affected genes were shown as the most upregulated (up), downregulated (down), and overall (total) effected. The top-10 terms in each category, including cellular component, biological process, as well as molecular function, are listed in the column charts.

GO analysis was carried out to determine the biological function of DEGs in dairy cow mammary glands, which were enriched by 913 GO terms (*p* < 0.05). The top ten terms in each category, including cellular component, biological process, as well as molecular function, are listed in the column charts (Figure 3). The result showed that the most affected functional category was related to immune response, including antigen processing (GO: 0002476), T-cell-mediated cytotoxicity regulation (GO: 0001916), acute-phase response (GO: 0006953), and immune response (GO: 0006955). Most of those immunology-related terms in which HS downregulated the expression of genes were also the top terms in the category. On the other hand, most of the terms in which heat stress upregulated the expression of genes were involved in heat shock response, such as chaperone-dependent refolding of protein (GO: 0051085), inflammatory response (GO: 006954), and heat shock protein (HSP) binding activity (GO: 0031072).

### 3.5. Biological Pathway Affected by HS

**Figure 4:**
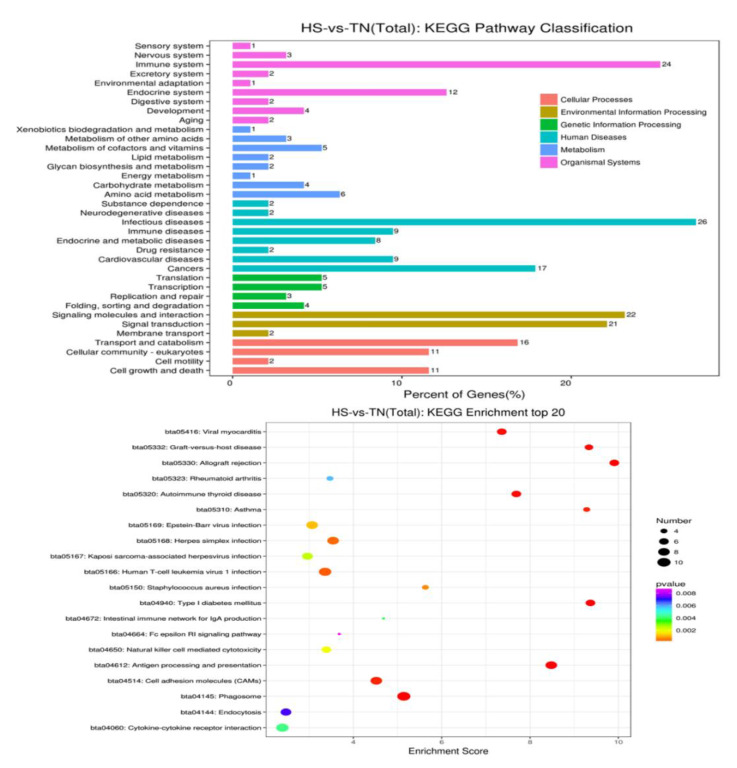
The top-20 pathways of HS-vs.-TN (total) of Kyoto Encyclopedia of Genes and Genomes enrichment.

To get a better insight into biological function, the GO information of the DEGs were further analyzed with the help of the KEGG database. The evaluation and comparison of the 20 different most-affected pathways were filtered out via *p*-value significance (Figure 4 and Supplementary Materials Figure S1). Nearly all these top-affected pathways could be related to the immune and heat shock response mentioned in Table 5. Six pathways are associate with exogenous pathogen invasion, including viral myocarditis, Epstein-Barr virus infection, *Herpes simplex* infection, *Staphylococcus aureus* infection, leishmaniasis, and antigen processing and presentation. In addition, four of them have a very close relationship to an autoimmune disorder, including rheumatoid arthritis, autoimmune thyroid disease, asthma, type I diabetes mellitus asthma, and all of them are associated with cellular responses by heat stress, including antigen processing and presentation, protein processing in the endoplasmic reticulum, and spliceosome. Therefore, all the results are directly or indirectly linked with immune regulation. 

### 3.6. Identification of Differentially Expressed Genes in Response to HS Group

**Figure 5:**
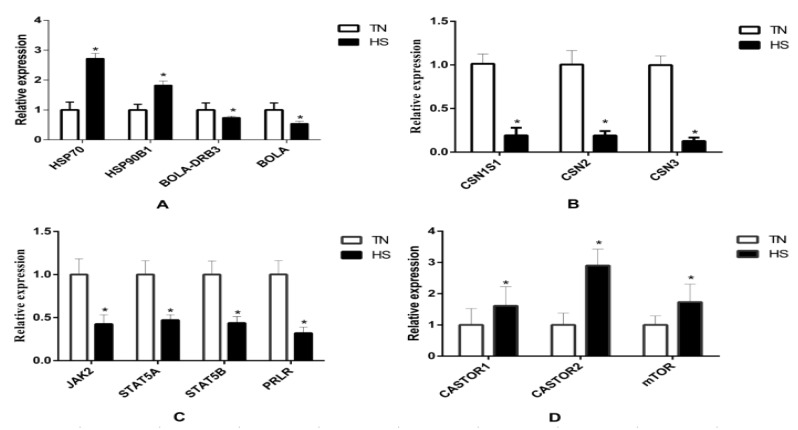
The relative expression of heat shock and immune response genes (**A**), casein genes (**B**), *JAK/STAT5* pathway genes (**C**), and *mTOR* pathway genes between both TN and HS groups (**D**). * *p* < 0.05 vs. TN group.

**Figure 6:**
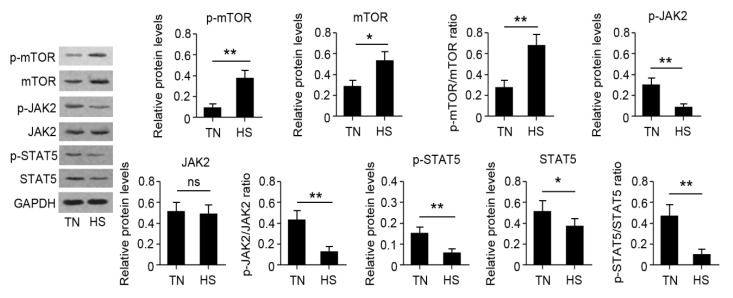
Identification of differentially expressed protein by Western blot assay (WBA). The WBA was carried out to find the protein and phosphorylation levels. * *p* < 0.05 or ** *p* < 0.01 vs. TN group. Ns: no significance.

The results of qRT-PCR are presented in Figure 5. Results revealed that fourteen genes were significantly affected by HS. RNA-seq results showed that *HSP70* and *HSP90B1* were upregulated while BoLA and BoLA-DRB3 were downregulated in the HS group. Moreover, HS decreased the gene expression of *CSN1S1*, *CSN2*, *CSN3*, *PRLR*, *STAT5A*, *STAT5B*, and *JAK2*, while HS increased the gene expression of *CASTOR1*, *CASTOR2*, and *mTOR*. The change was also consistent with that of their total protein products (Figure 6). The phosphorylation of *mTOR* was increased while the phosphorylation of *JAK2* and *STAT5* was decreased in the HS group.

## 4. Discussion

In intensive management systems, THI has been widely used as vital indicator to assess HS in dairy production, and it is generally recognized that when THI is above 68, dairy cows experienced HS [21]. In the current study, it was observed that the averaged THI in the HS group was ranged from 79.0 to 83.2 (Figure 1), which was much higher than the minimum THI (THI = 68) needed to induce HS in cows. Therefore, it could be assumed that cows in the HS group suffered from heat stress. It has been reported that milk yield drops sharply when THI is above 69 [22]. In the present study, the reduction of 16% milk yield could be related to higher THI in HS group cows. Respiratory rate and RT are the most imperative physiological indexes for evaluating the occurrence of HS in lactating cows [23,24]. It has been reported that heat stress rapidly enhances the lactating dairy cows’ RT and respiration rate [25]. Moreover, it has been reported that higher respiration rates and RTs are signs of HS for lactating dairy cows [26]. In the current study, higher respiration rates and RTs in the HS group further confirmed that the HS group cows experienced heat stress in the current experiment. Several study reports have suggested that heat stress persuades cell apoptosis [13], disturbs the normal biological activity of BMECs [12,13], and provoked intracellular thermotolerance responses of BMECs [10]. Furthermore, HS inhibited the transcription and translation of RNA in BMECs [14,27], especially the gene expression of *CSN1S1* [12,13]. In the current experiment, casein-coding genes were downregulated in the animals of the HS experimental treatment group as compared to the animals of the TN experiment treatment group. Findings of the current experiment are in line with the findings of researchers who have reported that at the cellular level, heat stress adversely affects the function and gene expression of casein in BMECs [10,11,12], especially the Alpha casein S1 (*CSN1S1*) [13]. Downregulation of casein-coding genes in the current study could be another reason for decreased milk production in HS group cows. 

HSPs function as molecular chaperones and are the most recognized cellular responses by heat stress. HSP70 is an abundant and sensitive acute protein during heat stress periods [28] and known as a reliable indicator of harsh environmental stress [29,30]. HSP70 also plays an important role in conferring thermo-adaptability and high levels of heat resistance of cells [31]. In the present experiment, an enhanced concentration of HSP70 in plasma was found in cows from the HS group. Furthermore, gene expression of *HSP90B1* and *HSPA1A* (*HSP70*) in mammary gland tissue were also upregulated in cows of the HS group. These results are similar to the results of previous researchers who have reported that the gene expression and synthesis of HSP (such as *HSPA1A*, *HSP90*, *HSP27*) were elevated when the cows suffered heat stress [11,12,32]. Moreover, GO enrichment analysis showed that *HSP90B1* and *HSPA1A* (*HSP70*) genes were involved in chaperone-dependent refolding of proteins (GO: 0051085) and HSP binding activity (GO: 0031072). It has been reported that the endogenous protein stores might have alternative fates during heat stress, and the synthesis of HSP70 during harsh weather may reduce the presence of circulating amino acids for synthesis of milk protein [33]. Therefore, when dairy cows experience heat stress, their cells produce a large number of HSPs, improperly folded protein, and unfold protein, which could be one of the factors contributing to the decrease in synthesis of milk protein. The results of blood glucose in the present study are in line with previous reports that have revealed that plasma glucose concentration was decreased by heat stress [34,35]. Heat stress could attenuate the response of the immune system significantly through effectively suppressing the blood glucose in dairy cows [36]. In vitro studies have suggested that heat stress weakens the functionality of immune cells isolated from thermal-neutral dairy cattle, including the ability to migrate and proliferate, phagocytose, and kill [37,38]. In our study, a GO enrichment analysis result showed that most of the affected functional category were related to immune response, such as antigen processing (GO: 0002476), T-cell-mediated cytotoxicity regulation (GO: 0001916), acute-phase response (GO: 0006953), and immune response (GO: 0006955). These results indicate a possible severe compromise in the immune system after the dairy cows experience heat stress.

The KEGG pathway analysis found that almost all of the top-affected pathways were related to immune response. Many genes are involved in those pathways, including *HSPA1A* (*HSP70*), *HSP90B1*, *BoLA*, *BoLA-DYA*, *LOC512672*, *LOC524810*, and *BoLA-DRB3*. A unique and most vital observation of this study is that the antigen processing and presentation pathway (bta04612) was significantly affected in HS group cows (Supplementary Materials Figure S2). In this pathway, the expression of the major histocompatibility complex (MHC) genes (*BoLA*, *BoLA-DYA*, and *BoLA-DRB3*) was downregulated while the gene expression of *HSPA1A* and *HSP90B1* was elevated. Previous studies have also reported that in hot environmental conditions, *HSP90* mRNA expression was significantly higher in Sahiwal, Tharparkar, and Murrah buffalo [39], Frieswal cattle [40] and goats [41]. It has been reported that HSP90B1 is the most available glycoprotein in the cell endoplasmic reticulum [42], and it is involved in the transport and processing of excreted proteins [42,43]. *HSP90B1* has been associated as an essential immune chaperone to regulate both adaptive and innate immunity [44]. It has also been reported that *HSP90B1* is necessary for the start of the innate immune response in animals [45]. Thus, it could be speculated that the enhanced expression of *HSP90B1* in heat stressed cows of the current study is related to the innate immune response.

The MHC genes like BoLA have received attention because of their relationship with the induction and regulation of immune responses of dairy cows [46]. Their function is to present foreign antigens (such as *Staphylococcus aureus*), after intracellular processing, to T-cells for a successful immune response [47]. It was reported that class II positive leukocytes specifically respond to local infusion of *Streptococcus uberis* in the bovine mammary gland [48,49,50], which could be related to clinical mastitis of dairy cows [51]. In the current experiment, we found that the expression of *BoLA* and *BoLA-DRB3* was downregulated in the pathway of antigen processing and presentation and the pathway of *Staphylococcus aureus* infection (bta05150), suggesting that the infection rate of cows was elevated in HS group due to exposure of heat stress. These findings are similar to the reports of earlier researchers who reported that dairy cows had a higher infection rate of *Staphylococcus aureus* and *Corynebacterium pseudotuberculosis*, a higher rate of clinical mastitis, and higher milk SCC when exposed to hot environments [51,52]. 

Both the acquired and innate immune responses can identify parts of pathogens called pathogen-associated molecular patterns, such as peptidoglycan bacterial DNA and LPS [53]. We observed that the cows in the HS experimental treatment had an expressively high level of LPS compared to the cows in the TN experimental treatment. The higher blood level of LPS and LPS-binding protein in the HS group animals suggest that heat stress activates a systemic inflammatory response [54]. It was reported that infusion of LPS intravenously in cows activated the immune system and resulted in the consumption of more glucose within 12 h after infusion, and it was concluded that immune activation redirects available nutrients from production to the immune system [55]. Therefore, the decrease of plasma glucose concentration and the increase of LPS concentration, along with the upregulation of *HSP90B1* gene expression, and the downregulation of *BoLA* and *BoLA-DDR3* gene expression in HS cow mammary gland tissues of the current experiment, means that exposure to heat activates the inflammatory responses and immune system, which redirect the nutrients from production to the dairy cows’ immune system.

It has been reported that prolactin (PRL) is correlated with hormones such as hydrocortisone and insulin during the lactation periods of cows [56] and known to turn on milk protein gene expression [57] via STAT5 phosphorylation by the PRLR and JAK2 [56,58]. The importance of STAT5 and PRLR in lactation has been shown in knockout mice in a previous study; it was reported that the removal of one of these proteins results in diminished mammary gland development and lactation [59]. It has also been reported that suppressors of cytokine signaling (SOCS) proteins and cytokine-inducible SH2-containing proteins (CIS) compose of a family of intracellular proteins [60] that are stimulated by PRL, act through feedback to inhibit cytokine signaling [61], and regulate the responses of immune cells to cytokines [60]. In particular, SOCS-1 and SOCS-3 have been shown to bind to cytokine receptors or to receptor-associated Janus-associated kinases to inhibit the activation of signal transducers and activators of transcription members, and ultimately interferon signaling [62]. Interestingly, in our study, the Jak/STAT5 pathway members, like JAK2, PRLR, STAT5A, and STATB, were also downregulated in the HS group, implying that exposure to heat could suppress the JAK2/STAT5 pathway to reduce milk production and suppress immune response. Therefore, it could be assumed that heat exposure to cows affects the immune function through changes in the PRL signaling pathway. 

Previous studies on cows and mice have reported that amino acids, like leucine, isoleucine, methionine, and threonine, act as signaling molecules and positively regulate milk synthesis and lactation [63]. The milk synthesis and lactation regulation are generally believed to be conducted through amino acids sensors like vacuolar H^+^-ATPase, SLC38A9, Sestrin2, CASTOR1 homodimer, and CASTOR1-CASTOR2, which serve as upstream activators of mTORC1 [64]. After being activated, the mTOR pathway regulates its targets to promote milk protein synthesis [65]. It has been reported that bovine mammary cells, which were incubated in high temperature (42 °C), reduced protein translation by reducing the mTOR downstream pathway activity [66,67]. In contrast to the findings of Kaufman et al. [66] and Salama et al. [67], we observed that heat exposure to cows enhanced the gene expression of *CASTOR1*, *CASTOR2* and phosphorylation of mTOR. However, findings of the present experiment are similar with the results of previous researchers, who stated that mTOR is negatively related to milk production [68]. It has been further reported that mTOR is one of the most classic autoimmune response regulators [69,70], and we also found that several autoimmune-related pathways were found to be significantly changed by HS exposure, which suggests a tight connection among the increased mTOR, reduced milk production, and immune response. 

## 5. Conclusions

The current study employed the RNA-seq technology to analyze the impact of HS on the whole transcript sequencing profile in the mammary glands of lactating Holstein dairy cows. The results of the current study reveal that heat stress downregulates the expression of casein genes, which result in a decrease in milk production. Moreover, according to functional annotation analysis, heat stress upregulates the gene expression of *HSPA1A* and *HSP90B1*, while iy downregulates the expression of immune response-related genes (*BoLA* and *BoLA-DRB3*) that eventually affected the immune function of the dairy cows and resulted in a reduction in milk yields. Furthermore, the results also reveal that under heat stress, the synthesis of heat shock and unfolded proteins was increased, which could reduce the accessibility of circulating amino acids for milk protein synthesis. Findings of current experiment may help to explore the impact of heat stress on immune function, milk production, and milk protein synthesis in cows.

## Figures and Tables

**Table 1:** Characteristics of the experimental cows.

Parameter	TN (thermal neutral)	HS (heat stress)	*p*-Value
Number of cows	10	10	
Parity	2.1 ± 1.0	2.2 ± 1.3	0.89
Lactation days	130.5 ± 15	123.4 ± 20	0.92
305-day milk yield (kg)	8993.7 ± 767.5	8933.2 ± 757.1	0.95
Average bodyweight (kg)	605.8 ± 58.1	603.5 ± 45.3	0.97

**Table 2:** Ingredients and chemical composition of the basal diet.

Ingredients (% of DM)	Content
Corn	14.94
Soybean meal	4.98
Cottonseed meal	1.30
Rapeseed meal	0.42
Extruded full-fat soybean	2.21
Corn gluten meal	0.83
Dried distillers grains	1.11
Oat hay	5.03
Alfalfa hay	10.75
Whole cottonseed	3.42
Whole corn silage	38.87
Beet pulp	2.52
Molasses	3.42
Fatty power	0.57
Brewer’s grains	6.86
Limestone	0.77
Dicalcium phosphate	0.42
Vitamin–mineral premix ^1^	0.48
Sodium bicarbonate	0.70
MgO	0.14
NaCL	0.28
Chemical composition (%)	
NE_L_ (Mcal/Kg) ^2^	1.70
Crude protein	16.6
Ether extract	5.50
NDF ^3^	30.89
ADF ^4^	19.27
Ca	0.78
P	0.43
Ash	7.50

^1^ Provided TMR/kg: a min. of 10,000 IU of vitamin A.; 1850 IU of vit. D; 50 IU. of vit. E; 16.73 mg of niacin; 54 mg of Zn; 12.5 mg of Cu; 0.45 mg of Se; 20.5 mg of Mn; 0.54 mg of Co; 0.945 mg of I. ^2^ Calculated. following NRC (2001) recommendation and was, based on actual DMI.. ^3^ Neutral detergent fiber. ^4^ Acid detergent fiber.

**Table 3:** Respiratory rate and rectal temperature of lactating Holstein cows in TN and HS groups.

Table	Treatment		SEM	*p*-Value
	TN	HS		
Respiration rate (breath/min)				
0700	35.6	72.4	2.51	0.03
1400	39.3	89.6	3.31	<0.01
2000	41.3	84.3	4.32	<0.01
Rectal temperature (°C)				
0700	38.2	39.2	0.08	<0.01
1400	38.5	39.5	0.09	<0.01
2000	38.4	39.3	0.07	<0.01

**Table 4:** Milk yield, dry matter intake, milk composition, and somatic cell count of cows in the TN and HS groups.

Items	Treatment		SEM	*p*-Value
	TN	HS		
Milk yield (kg/d)	42.5	35.6	2.03	<0.01
DMI (kg/d)	23.5	21.4	0.10	<0.01
Milk Protein %	3.2	2.8	0.07	0.03
Milk protien yield (kg/d)	1.3	1.0	0.06	0.12
Milk Fat %	4.3	3.8	0.14	0.04
Milk Lactose %	4.6	4.7	0.03	<0.01
UN of milk (mg/dL)	13.9	14.6	0.86	0.51
LPS (EU/L)	691.4	948.1	72.81	0.01
HSP 70 (ng/mL)	7.8	14.0	1.31	<0.01
Glucose (mm/L)	2.8	2.2	0.22	0.03
NEFA (μm/L)	167.0	238.2	18.51	0.02
SCC 1000/ML	266.0	307.1	132.46	0.76

**Table 5:** Significantly enriched KEGG pathways of downregulated and upregulated genes.

Term	*p*-Value	Genes	Gene Expression
bta05416, Viral myocarditis	<0.01	*BoLA*; *BOLA*; *BOLA-DRB3*; *BOLA-DYA*; *LOC512672*; *LOC524810*	Down
bta05169, Epstein-Barr virus infection	<0.01	*BoLA*; *BOLA*; *BOLA-DRB3*; *BOLA-DYA*; *LOC512672*; *LOC524810*	Down
bta05168, Herpes simplex infection	<0.01	*BoLA*; *BOLA*; *BOLA-DRB3*; *BOLA-DYA*; *LOC512672*; *SP100*	Down
bta05150, Staphylococcus aureus infection	<0.01	*BOLA-DRB3*; *BOLA-DYA*; *LOC524810*	Down
bta5140, Leishmaniasis		*BOLA-DRB3*; *BOLA-DYA*; *LOC524810*	Down
bta04612, Antigen processing and presentation	0.001	*BoLA; BOLA*; *BOLA-DRB3*; *BOLA-DYA*; *LOC512672*	Down
bta05323, Rheumatoid arthritis	0.003	*BOLA-DRB3*; *BOLA-DYA*; *LOC524810*	Down
bta05320, Autoimmune thyroid disease	<0.01	*BoLA*; *BOLA*; *BOLA-DRB3*; *BOLA-DYA*; *LOC512672*; *LOC524810*	Down
bta05310, Asthma	<0.01	*BOLA-DRB3*; *BOLA-DYA*; *LOC524810*; *MS4A2*	Down
bta04940, Type I diabetes mellitus	<0.01	*BoLA; BOLA*; *BOLA-DRB3; BOLA-DYA*; *CPE*; *LOC512672*	Down
bta04612, Antigen processing and presentation	0.001	*HSPA1A*; *HSPA8*; *LOC618733*, *HSP90B1*	Up
bta04141, Protein processing in endoplasmic reticulum	0.002	*DNAJA1*; *HSPA1A*; *HSPA8*; *HSPH1*, *HSP90B1*	Up
bta03040, Spliceosome	<0.01	*HSPA1A*; *HSPA8*; *PHF5A; SNRPG*	Up

## Supplementary Materials

The following are available online at https://www.mdpi.com/2076-2615/10/6/1015/s1. Figure S1: The top-20 pathway of the up and down pathway of KEGG Enrichment between HS and TN dairy cows, Figure S2: The pathway of antigen processing and presentation—*Bos taurus* (cow). The genes in the red box are significantly regulated by heat stress, Table S1: The primer information in the PCR assay, Table S2: The results of sequencing data quality preprocessing.
