# Supplementary material for: Transcriptome Functional Analysis of Mammary Gland of Cows in Heat Stress and Thermoneutral Condition
Source: Animals (Basel). 2020 Jun 10;10(6):1015. doi: 10.3390/ani10061015 (PMC7341491; doi:10.3390/ani10061015)
Supplement: Supplementary file 1 [file animals-10-01015-s001.pdf]

Supplementary Materials:

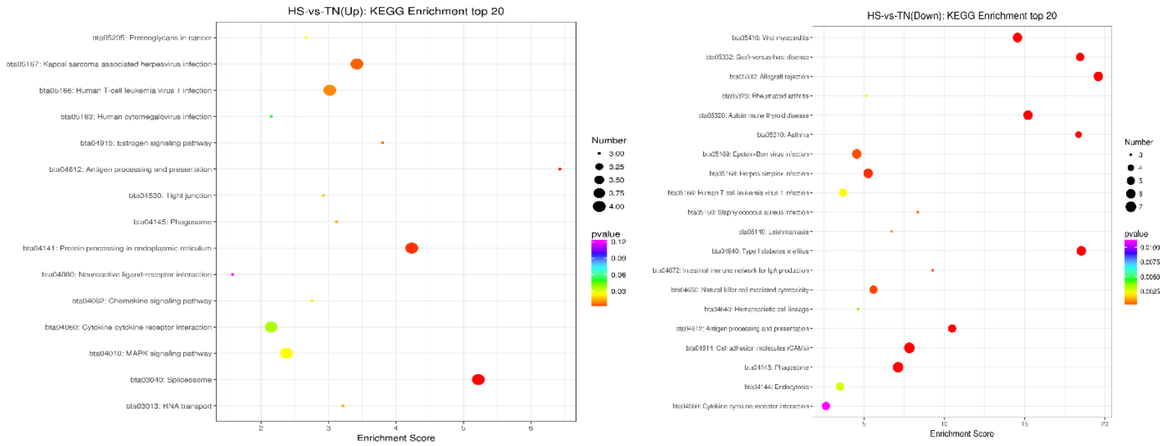

(**a**) (**b**)

Figure S1. The Top 20 pathway of the up and down pathway of KEGG Enrichment between HS and TN dairy cows. (**a**). The top-20 pathways of HS-vs.-TN (UP) of KEGG enrichment. (b). The top-20 pathways of HS-vs.-TN (Down) of KEGG enrichment.

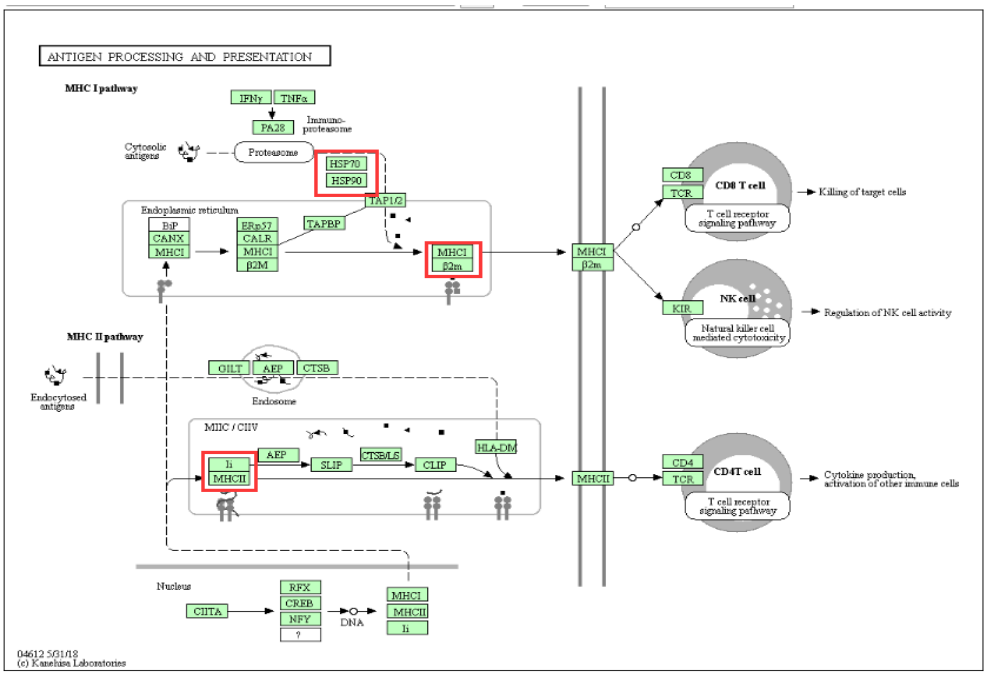

Figure S2. The pathway of antigen processing and presentation—*Bos taurus* (cow). The genes in the red box are significantly regulated by heat stress.

Table S1. The primer information in PCR assay.

Gene	Accession	Primers	Size/bp	TM(℃)
CSN1S1	XM_024993016	F: AATCCATGCCCAACAGAAAG	189	56.3
		R:TCAGAGCCAATGGGATTAGG		56.7

CSN2	XM_015471671	F: AGCCTCTTCCTCCAACTGTC	107	59
		R: ATCTCTCTGGGGATAGGGCA		59.1
CSN3	NM_174294	F: GGCGAGCCTACAAGTACACCTA	106	61.5
		R: GGACTGTGTTGATCTCAGGTGG		60.6
JAK2	XM_024996130	F:TGAAGAAAACAGGTAATCAGACTGGA	101	60.2
		R: AACATTTTCTCGCTCAACAGCA		59.4
STAT5A	NM_001012673	F: GCAGCTCCAGAACACGTACG	101	61.3
		R: CATTGTTGGCTTCTCGGACC		59.2
STAT5B	XM_005220675	F: TCCTGGATGACGAGCTGATC	115	58.9
		R: ATCTCCGCCAACTTCTCACA		59
HSP90B1	NM_174700	F: ATCGAGAAGGCTGTGGTGTC	88	59.8
		R: TCTCCATGTTGCCAGACCAC		59.9
HSPA1A	NM_203322	F: AGGACTTCGACAACAGGCTG	141	60
		R: TGCTGGACGACAAGGTTCTC		60
CASTOR1	XM_005218049	F: TTCCAGCAACGGCTTTCCC	94	60.9
		R: CAGCGTGAGGACGCAGAATC		61.4
CASTOR2	XM_002698179	F:ATCCCGCTCTTTACCTACGGC	150	62
		R: CAGGTGTTCCGAAGAGGGCA		62.1
PRLR	XM_024981207	F: ATAGCATGGTGACCTGCATCC	91	60.2
		R: TCTTCGGACTTGCCCTTCTC		59.4
mTOR	XM_002694043	F:CGTTCCTCTCAACATGGACACA	102	60.5
		R:AGCTTCTCCGCGTCTTTACAA		60.3
BOLA-DRB3	NM_001012680	F:GCCAAGTGGATCACCCCAAG	96	69.7
		R:CCCCGACTCCACTCATCATC		59.6
GAPDH	NM_001034034	F:GGGTCATCATCTCTGCACCT	176	55.3
		R: GGTCATAAGTCCCTCCACGA		55.3

Table S2. The results of sequencing data quality preprocessing.

Sample	Raw_Reads	Raw_Bases	Clean_Reads	Clean_Bases	Valid_Bases	Q30	GC
Sample_4025	49.34M	7.40G	48.20M	7.03G	95.04%	95.61%	47.37%
Sample_4555	49.16M	7.37G	47.95M	6.97G	94.49%	95.27%	48.76%
Sample_565	49.16M	7.37G	48.06M	7.01G	95.00%	95.65%	47.51%
Sample_6289	49.37M	7.40G	47.95M	6.97G	94.17%	95.54%	47.06%
Sample_6634	49.77M	7.47G	48.76M	7.05G	94.49%	96.04%	46.90%
Sample_6729	48.98M	7.35G	47.40M	6.86G	93.32%	94.63%	49.24%
